# Cannabinoid exposure in infants and children in the pediatric emergency department—the child protection perspective

**DOI:** 10.1007/s00431-025-06129-1

**Published:** 2025-04-24

**Authors:** Dvora Fridler, Lea Ohana Sarna Cahan, Adi Bracha Moshe, Noa Guzner, Itai Gross, Saar Hashavya

**Affiliations:** 1 Department of Pediatric Emergency Medicine, Hadassah-Hebrew University Medical Center, Jerusalem, Israel; 2 Department of Pediatrics, Hadassah-Hebrew University Medical Center, Jerusalem, Israel

**Keywords:** Cannabinoid exposure, Pediatric emergency department, Child protection, Urine toxicology, Cannabis intoxication, Child protection team (CPT)

## Abstract

Cannabinoid exposure in pediatric populations has become an increasing concern with the growing legalization and availability of cannabis products. While studies have addressed the clinical presentation of intoxication, limited data exist on child protection team (CPT) activation and child safety concerns in these cases. A 10-year retrospective study (2010–2021) was conducted at Hadassah Medical Center who operates two campuses within the Jerusalem metropolitan area. Cases of children aged 0–48 months with positive urine toxicology for cannabis were analyzed. Data collected included demographics, clinical presentation, and involvement of CPT, child protection services, and police. Out of 36 cases, 29 met the inclusion criteria. The majority (65.5%) were male, with a median age of 14 months. Neurological symptoms were universal, and CPT was activated in all cases (100%). Police reports were filed in 93.1%, while child protection services were contacted in 62.1%. Despite positive drug screens, only one caregiver admitted to cannabis exposure, and more than 60% denied any knowledge of exposure. Most children had no prior reports with social services; however, 11 (37.9%) had multiple pediatric emergency department (PED) visits, suggesting a heightened risk of neglect. *Conclusions*: Cannabis intoxication in young children presents diagnostic challenges due to non-specific symptoms and low caregiver disclosure rates, often leading to unnecessary investigations. Although prior reports of abuse or neglect are uncommon, frequent PED visits may signal neglect. Early CPT involvement is essential in managing these cases and ensuring child safety.

**Table:** 

**What is known:** *• Pediatric cannabis intoxication presents primarily with non-specific neurological symptoms, making early diagnosis challenging.* *• Most existing studies focus on the clinical aspects of intoxication, with limited data on child protection team involvement and reporting practices.*	
**What is new:** *• Universal involvement of the Child Protection Team (CPT) led to high reporting rates to authorities, with nearly all cases being reported either to the police or to Child Protection Services.* *• Multiple pediatric emergency department visits, especially for injuries, may indicate neglect even when prior CPS reports are absent.*

**What is known:**

*• Pediatric cannabis intoxication presents primarily with non-specific neurological symptoms, making early diagnosis challenging.*

*• Most existing studies focus on the clinical aspects of intoxication, with limited data on child protection team involvement and reporting practices.*

**What is new:**

*• Universal involvement of the Child Protection Team (CPT) led to high reporting rates to authorities, with nearly all cases being reported either to the police or to Child Protection Services.*

*• Multiple pediatric emergency department visits, especially for injuries, may indicate neglect even when prior CPS reports are absent.*

## Introduction

Cannabis use has become increasingly prevalent worldwide, with many countries promoting legalization [1–3]. Children’s unintended ingestion of cannabinoids has emerged as a significant concern, particularly in light of the increased availability of cannabis products as a result of legalization and increased medical use [1–3].

Cannabis is available in various forms, including edible products such as chocolates, gummies, brownies, and hashish, as well as in cigarette or joint form for smoking. Importantly, edible cannabis products have been associated with a heightened incidence of exposure and intoxication in pediatric populations [1, 2, 4].

Previous studies have focused on the many and varied manifestations of cannabis intoxication that can be expressed in neurological, respiratory, and cardiovascular symptoms [3, 5–8].

While most studies have described the clinical presentation of pediatric cannabinoid intoxication, there is a lack of data on its social aspects, the risk of abuse and neglect, and child protection team (CPT) and service (CPS) activation patterns in these children. Pellisier et al. presented a series of 12 children with cannabis intoxication, of whom nine were referred to social services (CPS) [5]. A recent study [4] of 179 children found that 86% were reported to the CPS in the community; however, hospital CPT activation was not reported.

This retrospective study addressed the presentation and management of cannabis intoxication in children with a specific emphasis on social aspects and child protection concerns.

## Methods

In this retrospective study, charts of children who presented to the pediatric emergency department (PED) at Hadassah Medical Center for cannabinoid exposure were analyzed.

Data were collected from electronic medical records (EMRs) and ancillary tests between 2010 and 2021 for all children aged 0–48 months who were referred to or admitted to Hadassah Medical Center for suspected cannabinoid ingestion. Inclusion criteria included children aged 0–48 months who underwent urine toxicology screening with a confirmed positive result for cannabinoids. Exclusion criteria included neonates of mothers with suspected cannabinoid consumption in the nursery or incomplete data. Specifically, cases were excluded if any of the following key data points were missing: demographics, clinical symptoms at presentation, and course of evaluation.

All children had their urine toxicology screening and work up performed in the PED; no cases of direct admission or urine toxicology screening were recorded in the pediatric department.

The children’s demographics, the symptoms noted upon their presentation, and the course of their evaluation and treatment were logged into their files. Child protection team involvement and reports to CPS and or the police were also recorded as well as previous CPS reports.

The CPT at Hadassah medical center consists of a social worker and a pediatrician available 24 h a day. Activation of the CPT may be partial and include social workers alone or combined to include both the social worker and the attending pediatrician. The decision to alert the child protection team rests on the attending nurse or physician in the PED.

CPT involvement included a complete physical examination conducted by a pediatrician to assess for signs of physical abuse, alongside a social work evaluation focused on gathering social history and identifying risk factors. Based on the findings of these assessments, the CPT determined whether additional investigations, such as skeletal surveys, Fundoscopy, or other imaging studies, were indicated.

In Israel, mandatory reporting laws require any individual who has a reasonable suspicion of child abuse or neglect to file a report. According to the law, the report may be submitted either to child protection services (CPS) or to the police, at the discretion of the reporter.

If deemed necessary, a report to the CPS or police is sent by the CPT.

The total number of ED visits for each patient, both prior to and following the cannabis-related presentation, was extracted from the complete medical record without applying a predefined time limit. In practice, the available records included ED visits that occurred between 2006 and 2023. The total number of ED visits was collected as a potential marker of child abuse or neglect, based on the assumption that frequent or repeated ED visits may indicate underlying concerns regarding the child’s safety and well-being.

The study was approved by the institutional review board (Approval #0615–22-HMO). Data collection was carried out in accordance with the ethical standards of the committee.

## Statistical analysis

Descriptive analyses were conducted; means and standard deviations (SDs) were calculated. Frequencies, percentages, proportions, and 95% confidence intervals were obtained.

## Results

### Demographics and clinical presentation(Table 1)

**Table 1:** Epidemiology and clinical course

	*n* (%)/SD	CI 95%
**Demography**		
Male	19 (65.5%)	
Mean age, months	13.08 (± 3.4)	11.7–14.3
**Symptoms**		
Neurological	29 (100%)	
Gastrointestinal	7 (24.1%)	
Respiratory	7 (24.1%)	
Cardiovascular	7 (24.1%)	
Mydriasis	7 (24.1%)	
Thermoregulation	7 (24.1%)	
**Workup**		
Blood tests	29 (100%)	
Lumbar puncture	6 (20.7%)	
Head CT*	6 (20.7%)	
**Hospitalization course**		
Admissions	27 (93.1%)	
PICU^ admissions	15 (51.7%)	
Length of stay, days (range)	2.59 (± 1.2)	2.13–3.05

^*^CT computed tomopgraphy, ^*PICU* pediatric intensive care unit

**Fig. 1:**
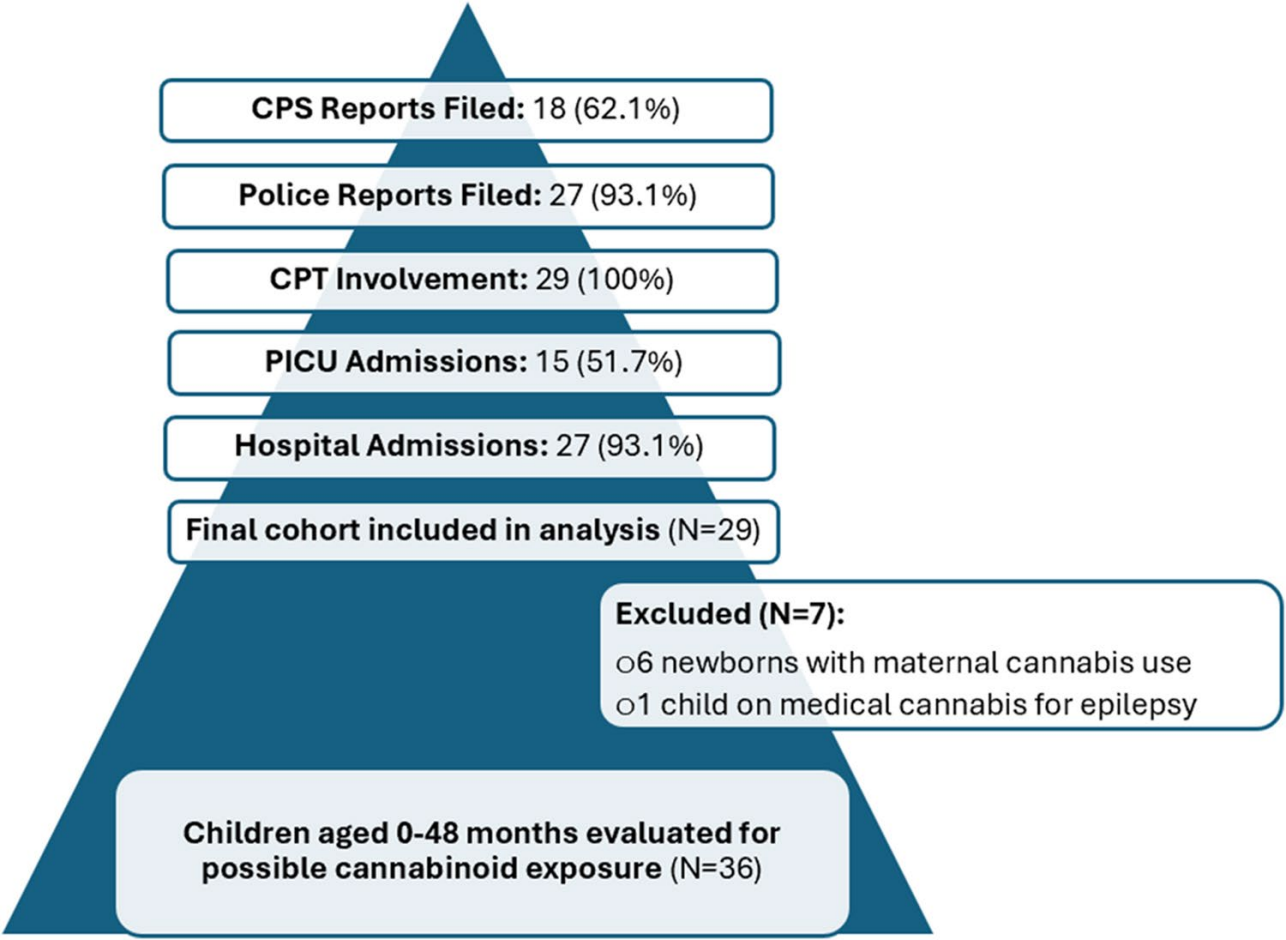
Flowchart for case selection. Thirty-six children met the initial inclusion criteria. Seven were excluded

A total of 36 positive urine drug tests for cannabinoids was recorded but 7 cases were excluded due to prior documented exposure to cannabis: six were newborns with mothers who tested positive for cannabis, and one was a child known to be treated with medical cannabis for epilepsy. Case selection flowchart is depicted in Fig. 1.

A final sample size of 29 children was available for analysis, of whom 19 (65.5%) were male, with a median age was 13.08 ± 3.4 (CI 11.7–14.3) months, with the youngest aged 8 months and the oldest aged 21 months.

Twenty-seven (93.1%) of the 29 children were admitted to the hospital, 15 (51.7% of admitted cases) required Pediatric Intensive Care Unit (PICU). The average length of stay in PICU was 0.6 ± 0.7 (CI 0.36, 0.95) days. The mean length of hospitalization was 2.56 ± 1.2 (CI 2.13, 3.05) days. No mortality was recorded.

Full disclosure of cannabis exposure by the caregivers occurred in only one case (3.4%), while 62.1% denied any known exposure even after receiving test results. In 6 cases (20.7%), the caregivers reported suspected substance ingestion outdoors (in the street or playground). In four cases (13.8%), suspected substance ingestion was reported indoors (at a relative’s or friend’s house); in 18 cases (62.1%), the source of the exposure was reported by the family to be unknown even after confronting the caregiver with the urine toxic test analysis results.

All 29 children were symptomatic at the time of presentation. Neurological symptoms were observed in all cases, with varying manifestations. The majority of the children, 22 (75.9%), exhibited lethargy, seizures were reported in five cases (17.2%), whereas two children (6.9%) displayed restlessness.

Seven children (24.1%) presented with mydriasis and 2 children (6.9%) displayed pinpoint pupils.

Gastrointestinal symptoms were reported in seven children (24.1%), with four (13.8%) experiencing vomiting and three (10.3%) having diarrhea. Respiratory symptoms were present in seven children (24.1%), six (20.7%) of whom had bradypnea and 1 (3.4%) with tachypnea. Constitutional symptoms were observed in seven children (24.1%), of whom 6 (20.7%) had fever and 1 (3.4%) hypothermia. Cardiovascular symptoms were reported in seven children (24.1%), all of whom presented with tachycardia.

All 29 (100%) children had venous blood laboratory tests taken, a lumbar puncture was performed in six (20.7%) children, and a head CT was performed in six (20.7%) children. All these tests were conducted before the positive urine test for cannabinoids was available.

No child was found to have a co-ingestion.

### Child protection team assessment and potential risk of child vulnerability (Table 2)

**Table 2:** Child protection aspects

	*n* (%)
**Source of exposure**	
Outdoors	6 (20.7%)
Indoors	5 (17.2%)
Unknown	18 (62.1%)
**CPT^ evaluation**	
Social worker	29 (100%)
Child abuse pediatrician	14 (48.3%)
**Report**	
Police	27 (93.1%)
Child protection services	18 (62.1%)
Previous report to CPS*	3 (10.3%)
**Multiple PED` visits**	
Total	11 (37.9%)
Injury related	6 (20.7%)
Various illnesses	5 (17.2%)

^*CPT* child protection team, *CPS child protection services, `*PED* pediatric emergency department

Activation of the CPT was recorded in all 29 (100%) cases. In 14 cases (48.3%), CPT activation included both the social worker and the pediatrician, whereas in the remainder, only the social worker’s report was documented.

Non-accidental injury assessment was performed in all children. One child was found to have bruises in the physical examination. In two (6.9%) children, a fundoscopy to assess retinal hemorrhages was performed with no pathological findings reported. None of the children underwent a skeletal survey, and no documentation was found regarding the indication or decision-making process for this assessment.

A report to the police was made in 27 (93.1%) of the cases, and a report to the CPS too was filed in 18 cases (62.1%). Only one case was not reported at all to the authorities (Table 2).

Three (10.3%) of the families of the patients had been previously reported to the CPS: two (6.9%) cases were related to economic difficulties and one case (3.4%) was associated with a history of parental incarceration. Twenty-six children (89.7%) came from low socioeconomic neighborhoods as determined by the municipal social services. Two children (6.9%) were from outside the Jerusalem metropolitan area, and only one (3.4%) was from a high socio-economic neighborhood. The mean number of children per family was 2.4 (± 1.6), and 11 (37.9%) were firstborn.

Among 29 children with a positive urine test, 11 (37.9%) had multiple visits to the PED. Among them, 6 (20.7%) had injury-related revisits, which may suggest possible neglect or abuse. Of these injury-related revisits, lacerations and minor head injuries were the most common diagnoses. Notably, CPT involvement was not documented in any of the revisits (Table 3).

**Table 3:** Revisit characteristics

Case number	Number of revisits	Injury-related visits	Age	Diagnosis	CPT involvement
Case 1	3	2	7Y4M	Minor head injury	No
			8Y2M	MVA*-pedestrian	No
Case 2	1	0			
Case 3	2	2	2Y11M	Open wound face	No
			6Y	Open wound face	No
Case 4	9	1	1Y9M	Minor head injury	No
Case 5	4	1	3Y	Open wound face	
Case 6	4	2	3 M	Minor head injury	No
			6Y4M	Open wound face	No
Case 7	2	0			
Case 8	1	0			
Case 9	5	0			
Case 10	1	1	2Y6M	Open wound face	No
Case 11	4	0			

^*CPT* child protection team, *MVA motor vehicle accident

## Discussion

In this study, encompassing 29 cases of pediatric cannabinoid intoxication, different features including epidemiology, clinical manifestations, diagnostic workup, and implications for child protection were investigated.

Although exploratory ingestion is often suspected, 62.1% of cases had an unknown source of exposure, indicating that the actual cause remains unclear in most cases.

As in other studies [6, 7, 9, 10] the findings revealed that the children were predominantly toddlers intoxicated as a result of exploratory ingestion. As reported in other studies, neurological symptoms were universally observed in all cases. The other affected systems included the gastrointestinal tract, the respiratory system, and the cardiovascular system [3, 7, 8].

While in previous studies, a history of cannabis exposure and place of exposure were reported in up to 49% [4]; in this study, cannabis ingestion on presentation was only disclosed in one case, leading to an extensive diagnostic workup in the other cases. Even after intentional anamnesis for potential cannabis exposure, caregiver disclosure of accidental cannabis ingestion was reported in only nine (34.5%) cases, leaving 18 cases (62.1%) with an unknown source of exposure, in contrast to other studies where unidentified exposure was reported in 19.3% [9] and 9% [7]. One plausible explanation for the low report rate of cannabis exposure is that the use of cannabis in Israel remains illegal apart for prescribed cannabis medical use. The increased use of ancillary tests such as computed tomography (CT), lumbar puncture (LP), and other laboratory blood tests might have resulted from the initial disclaimer of cannabis use, thus highlighting the importance of focused anamnesis and a prompt toxicological urine test in case of suspected intoxication. Workups were performed at the discretion of the attending physician, leading to differences in the extent of assessments conducted.

Few studies have specifically addressed the report rate to the hospital CPT and tend to focus instead on CPS reports, which can be as high as 75 to 86% [4, 5]. In this study, all cases were reported to the hospital CPT.

The CPT reported all the cases but one to the authorities. A report to the police was filed in 93.1% of the cases, and a report to the CPS too was filed in 62.1%. The rather low CPS reporting by the CPT might reflect the erroneous assumption that reporting to the police may suffice, robust protocols and increasing CPT awareness is suggested in order to enhance CPS reporting rate.

Non-accidental injury diagnosis work up for children with positive urine test was lacking and inconsistent in part due to the lack of defined protocol in case of cannabis intoxication like in other cases as femur fracture and intracranial bleeding; indeed, as seen in other intoxications, there is no robust protocol for the assessment of non-accidental injury, and the work up is expert dependent.

Most children came from families with low SES as determined by the municipal geographical analysis, and most were from small families. Of the children, 37.9% were the firstborn. Surprisingly sparse data are available on the relationship between the number of siblings and the risk of exploratory ingestion; however, studies have noted an association between larger family size and risk of accidents [11]. As cannabis consumption is more prevalent among young people, it might lead to unintentional exposure in children of younger families rather than in large families, as in other high vulnerability activities.

Our analysis of previous family history reports to the social services revealed that only a minority of the families had prior contact, indicating that many of these cases represented new instances of concern, or a lack of high risk for abuse and/or neglect. A noteworthy proportion (37.9%) of the children had multiple return visits to the PED, with a subset of cases (20.7%) attributed specifically for injury-related visits. Previous studies have documented a risk of 30–33% among abused children for previous medical visits [12, 13]. This suggests a potential correlation between cannabis intoxication and increased risk of harm due to insufficient parental supervision, thus warranting further attention to ensure the safety of these children; interestingly, in this study, there was no CPT involvement in any of the injury-related revisits, highlighting the need for a structured protocol in children with referral or previous history of intoxication.

## Limitations and conclusion

This study has some limitations. It consisted of a small, single-center study conducted, retrospectively. As such, the findings may not be fully generalizable to broader populations or settings. The small sample size over a 10-year period may be attributed to illegality of cannabis use in Israel, as well as to underreporting and under diagnosis. Additionally, caregiver reluctance to disclose exposure may have further contributed to the low number of identified cases.

Finally, data regarding the specific interventions or outcomes following reports made to CPS or law enforcement were not available. As these processes occur outside the hospital setting and are not routinely documented in medical records, we were unable to evaluate the subsequent protective actions or social services involvement following discharge.

Overall, the findings show that cannabinoid intoxication is challenging to diagnose in places where cannabis is illegal and substantial gaps in both reporting and assessing for non-accidental injury are present even in the presence of an active CPT. While previous reports on abuse or neglect are infrequent in these children, their revisit patterns might suggest a risk of neglect, thus highlighting the important role of early protocol-guided CPT involvement in cannabis intoxication.

## Data Availability

No datasets were generated or analysed during the current study.

## References

[1] Myran DT, Tanuseputro P, Auger N, Konikoff L, Talarico R, Finkelstein Y (2023) Pediatric hospitalizations for unintentional cannabis poisonings and all-cause poisonings associated with edible cannabis product legalization and sales in Canada. JAMA Health Forum 4(1):E225041. 10.1001/jamahealthforum.2022.5041 36637814 10.1001/jamahealthforum.2022.5041 PMC9857209

[2] Hammig B, Jones C, Haldeman S (2023) Pediatric poisonings associated with ingestion of marijuana products. J Emerg Med 64(2):181–185. 10.1016/j.jemermed.2022.12.025 36822984 10.1016/j.jemermed.2022.12.025

[3] Wang GS, Le LMC, Deakyne SJ, Bronstein AC, Bajaj L, Roosevelt G (2016) Unintentional pediatric exposures to marijuana in Colorado, 2009–2015. JAMA Pediatr 170(9):e160971. 10.1001/jamapediatrics.2016.0971 27454910 10.1001/jamapediatrics.2016.0971

[4] Dubinin A, Bialostozky M, Richardson A, Laub N (2024) Presentation, management, and child protective service reporting of children who test positive for cannabis in an emergency room setting. www.pec-online.com. Accessed 4 Jun 2024 10.1097/PEC.0000000000003145 38471748

[5] Pélissier F, Claudet I, Pélissier-Alicot AL, Franchitto N (2024) Parental cannabis abuse and accidental intoxications in children prevention by detecting neglectful situations and at-risk families. www.pec-online.com. Accessed 12 Jul 2023 10.1097/PEC.0000000000000288 25407034

[6] Whitehill JM, Harrington C, Lang CJ, Chary M, Bhutta WA, Burns MM (2019) Incidence of pediatric cannabis exposure among children and teenagers aged 0 to 19 years before and after medical marijuana legalization in Massachusetts. JAMA Netw Open 2(8). 10.1001/JAMANETWORKOPEN.2019.9456 10.1001/jamanetworkopen.2019.9456 PMC6704738 31418807

[7] Claudet I, Mouvier S, Labadie M et al (2017) Unintentional cannabis intoxication in toddlers. http://publications.aap.org/pediatrics/article-pdf/140/3/e20170017/1104495/peds_20170017.pdf. Accessed 12 Jul 2023 10.1542/peds.2017-0017 28808073

[8] Wang GS, Hoyte C, Roosevelt G, Heard K (2019) The Continued Impact of Marijuana Legalization on Unintentional Pediatric Exposures in Colorado. Clin Pediatr (Phila) 58(1):114–116. 10.1177/0009922818805206 10.1177/0009922818805206 30288992

[9] Richards JR, Smith NE, Moulin AK. Unintentional cannabis ingestion in children: a systematic review. Published online 2017. www.jpeds.com. Accessed 12 Jul 2023 10.1016/j.jpeds.2017.07.005 28888560

[10] Cohen N, Blanco LG, Davis A et al (2022) Pediatric cannabis intoxication trends in the pre and post-legalization era. Clin Toxicol 60(1):53–58. 10.1080/15563650.2021.1939881 10.1080/15563650.2021.1939881 34137352

[11] Bijur PE, Golding J, Kurzon M (1988) Childhood accidents, family size and birth order. Soc Sci Med 26(8):839–843. 10.1016/0277-9536(88)90176-1 3375855 10.1016/0277-9536(88)90176-1

[12] Thorpe EL, Zuckerbraun NS, Wolford JE, Berger RP (2014) Missed opportunities to diagnose child physical abuse. Pediatr Emerg Care 30(11):771–776. 10.1097/PEC.0000000000000257 25343739 10.1097/PEC.0000000000000257

[13] King WK, Kiesel EL, Simon HK (2006) Child abuse fatalities. Pediatr Emerg Care 22(4):211–214. 10.1097/01.pec.0000208180.94166.dd 16651907 10.1097/01.pec.0000208180.94166.dd

